# Exploring the potential of generative artificial intelligence in medical image synthesis: opportunities, challenges, and future directions

**DOI:** 10.1016/j.landig.2025.100890

**Published:** 2025-08-14

**Authors:** Bardia Khosravi, Saptarshi Purkayastha, Bradley J Erickson, Hari M Trivedi, Judy W Gichoya

**Affiliations:** Department of Radiology, Department of Orthopedic Surgery, Mayo Clinic, Rochester, MN, USA; Luddy School of Informatics and Computing, Indiana University, Indianapolis, IN, USA; Department of Radiology, Mayo Clinic, Rochester, MN, USA; Department of Radiology and Imaging Sciences, Emory University School of Medicine, Atlanta, GA, USA; Department of Radiology and Imaging Sciences, Emory University School of Medicine, Atlanta, GA, USA

## Abstract

Generative artificial intelligence has emerged as a transformative force in medical imaging since 2022, enabling the creation of derivative synthetic datasets that closely resemble real-world data. This Viewpoint examines key aspects of synthetic data, focusing on its advancements, applications, and challenges in medical imaging. Various generative artificial intelligence image generation paradigms, such as physics-informed and statistical models, and their potential to augment and diversify medical research resources are explored. The promises of synthetic datasets, including increased diversity, privacy preservation, and multifunctionality, are also discussed, along with their ability to model complex biological phenomena. Next, specific applications using synthetic data such as enhancing medical education, augmenting rare disease datasets, improving radiology workflows, and enabling privacy-preserving multicentre collaborations are highlighted. The challenges and ethical considerations surrounding generative artificial intelligence, including patient privacy, data copying, and potential biases that could impede clinical translation, are also addressed. Finally, future directions for research and development in this rapidly evolving field are outlined, emphasising the need for robust evaluation frameworks and responsible utilisation of generative artificial intelligence in medical imaging.

## Introduction

Generative artificial intelligence is a class of deep learning models capable of creating content that diverges from traditional discriminative models focused on interpretation or decision making. Generative artificial intelligence has seen rapid advancements over the past 3 years, with large language models gaining substantial public attention after the introduction of ChatGPT, a model trained on an extensive corpus of text to create coherent and realistic responses to user queries.^1^ Large language models have shown noteworthy capabilities in the understanding and generation of natural language, paving the way for more advanced multimodal models that combine textual, visual, and contextual understanding. These large multimodal models have the potential to aid various domains, including health care, by integrating data from different input streams. Notable examples of large language models in medicine are Med-PaLM and Med-Gemini, which have shown promising results in tasks such as answering medical questions, summarising medical documents, and suggesting potential differential diagnoses on the basis of patient symptoms and test results. In addition, Med-Gemma and MedImageInsight are models trained on different types of medical images including radiology images (eg, chest x-rays, mammograms, CT), as well as dermatology and ophthalmology images, which allow end users to interact with the model using both language and images (and are thus known as multimodal foundation models). These multimodal models provide unconventional visual question answering ability and are able to learn from a few examples to perform downstream classification tasks.^2,3^

Preliminary evidence suggests that generative artificial intelligence in the realm of visual content has made remarkable advancements with models such as DALL-E, Stable Diffusion, Sora, and Veo, which excel in generating realistic images and videos based on textual prompts.^4–6^ Although these models primarily process text as input, with some using images for conditioning purposes, their primary focus is on generating high-quality images. Seminal works published since 2022 in medical imaging have shown the potential of generative artificial intelligence in creating realistic medical images (synthetic data), suggesting new approaches for research and clinical applications.^7–10^

This Viewpoint provides a comprehensive overview of synthetic data in medical imaging and critically analyses the advancements, applications, and challenges of this field. To this end, various image generation paradigms are examined, with the intention to assess how these generative technologies are changing the landscape of medical imaging research. The potential of these models and their derivative synthetic datasets, particularly their ability to augment and diversify medical research resources, are explored, in addition to their benefits in terms of data augmentation, anonymisation, and modelling biological phenomena. Finally, the challenges of using synthetic data are discussed, including the need for rigorous evaluation metrics and ethical considerations, and potential research directions are proposed that could substantially benefit the field of medical imaging.

## Synthetic datasets

### Generative models

The field of synthetic data is still in its nascent stages, with no consensus on a single, universally accepted definition as yet. This absence of a clear definition has led to inconsistencies in how the term is used and interpreted across various contexts, which in turn can affect the reproducibility and transparency of research involving synthetic data.^11^ The Royal Society and The Alan Turing Institute put forth a working definition of synthetic data in 2022, as data that have been generated using a purpose-built mathematical model or algorithm, with the aim of solving a (set of) data science task(s).^12^ This proposed definition emphasises the functional and intentional aspects of synthetic data, focusing on its strategic application in tackling complex scientific challenges rather than simply mimicking the statistical properties of the original data.

The advancement of generative artificial intelligence introduces a new concept in data sharing, which we refer to as a model as a dataset. In this concept, generative models learn and store patterns and characteristics of the original data in their internal parameters (weights).^13^ These trained weights contain a compressed version of the key features and relationships of the training data. Unlike traditional dataset sharing, which involves transferring actual images, sharing model weights provides an efficient alternative that allows others to generate new synthetic images with properties similar to the original data. These synthetic datasets have been shown to closely resemble the source data and capture their distribution, including the relationship of different anatomical features and their correlation with different pathological processes.^8,9^

Two broad categories of generative models provide the ability to generate synthetic datasets: physics-informed and statistical models.

Physics-informed models are primarily rule-based approaches that incorporate domain-specific knowledge and physics principles through mathematical equations and explicit constraints to generate realistic and physically plausible data. Rather than learning the patterns directly from data, these models encode expert knowledge and known physics laws (eg, fluid dynamics, tissue biomechanics, or radiation physics) to simulate biological phenomena. These models have been applied successfully in medical imaging to simulate anatomical structures (such as a shape model of the femoral bone), physiological processes (such as blood flow dynamics in vascular structures), and medical interventions (such as simulating the distribution of the radiation dose in radiotherapy planning).^14^ Physics-informed models offer high fidelity and interpretability but might require extensive domain expertise and computational resources.

**Figure 1:**
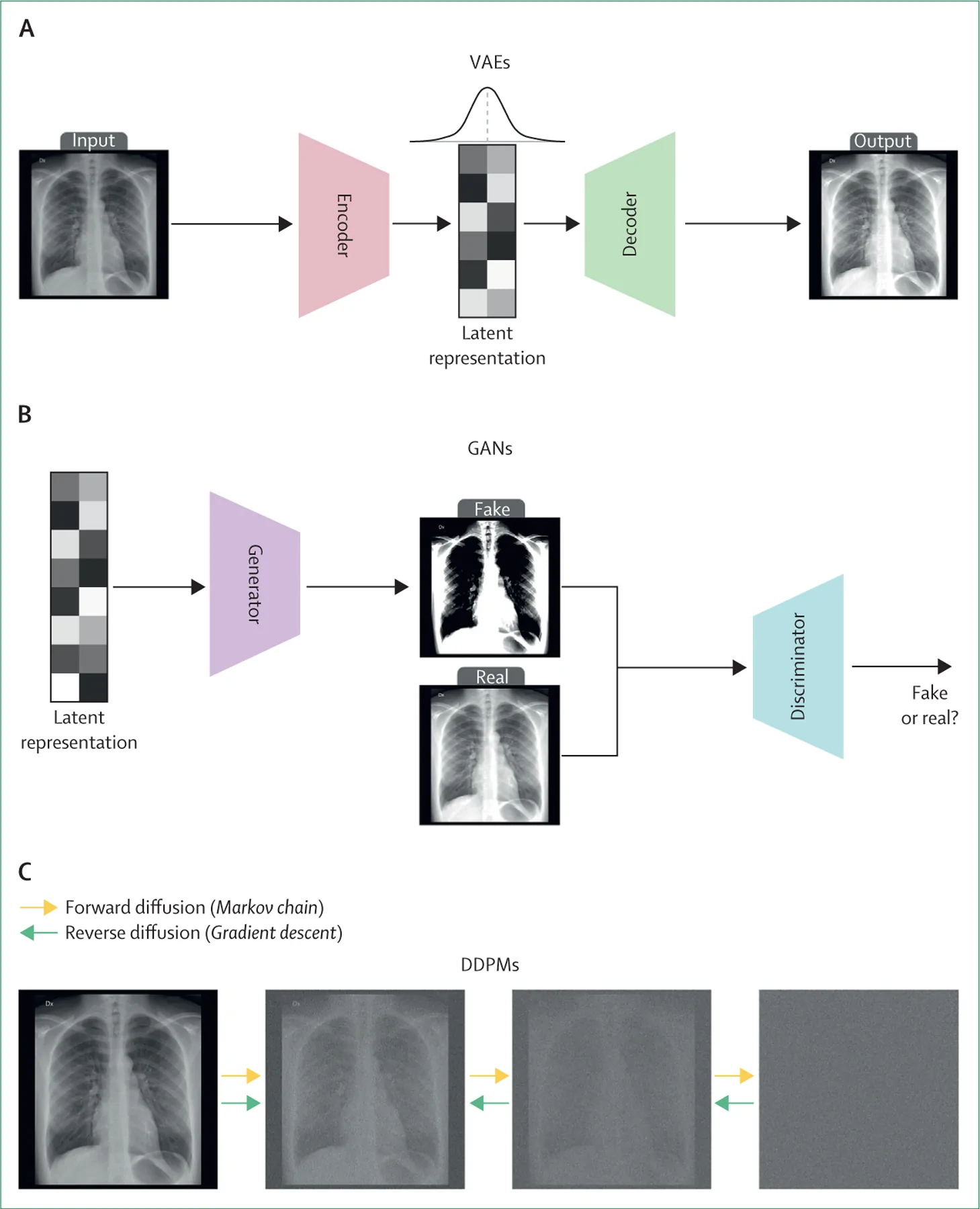
The architectures and key components of three popular statistical models used in image generation (A) VAEs consist of an encoder that compresses the input data into a lower-dimensional latent representation and a decoder that reconstructs the original data from the latent space. The model is trained to minimise the reconstruction error while also regularising the latent space to follow a previous distribution, typically a standard normal distribution. This training enables the generation of new samples by sampling from the learned latent distribution and decoding them. (B) GANs use a two-network architecture, with a generator that creates synthetic data samples and a discriminator that distinguishes between real and generated samples. The generator aims to produce samples that are indistinguishable from real data, whereas the discriminator provides feedback to guide the generator’s improvement. Through an adversarial training process, the generator learns to capture the underlying data distribution, enabling the creation of realistic samples. (C) DDPMs generate data by learning to reverse a noising process. The model starts with a sample from a simple distribution (eg, Gaussian noise) and iteratively denoises the sample using a learned Markov chain. At each step, the model estimates the gradient of the data distribution and refines the sample accordingly. By repeatedly applying this process, DDPMs can produce high-quality samples that closely resemble the training data. The figure depicts the forward diffusion process that gradually adds noise to the data and the reverse diffusion process that progressively denoises the sample to generate a clean output. DDPMs=denoising diffusion probabilistic models. GANs=generative adversarial networks. VAEs=variational autoencoders.

In contrast to physics-informed models, statistical models learn from data patterns and distributions (figure 1). Among them, variational autoencoders (VAEs) function by compressing data into a lower-dimensional representation, also known as latent space, and then reconstructing the data, thereby capturing the data distribution effectively.^15^ Generative adversarial networks (GANs) operate through a dual-network system, in which a generator creates data samples and a discriminator evaluates these data samples and provides feedback to the generator.^16^ This synergy continually enhances the quality and realism of the data generated. Denoising diffusion probabilistic models (DDPMs) introduce noise into an image and learn to reverse this process, producing high-quality samples.^17^

**Figure 2:**
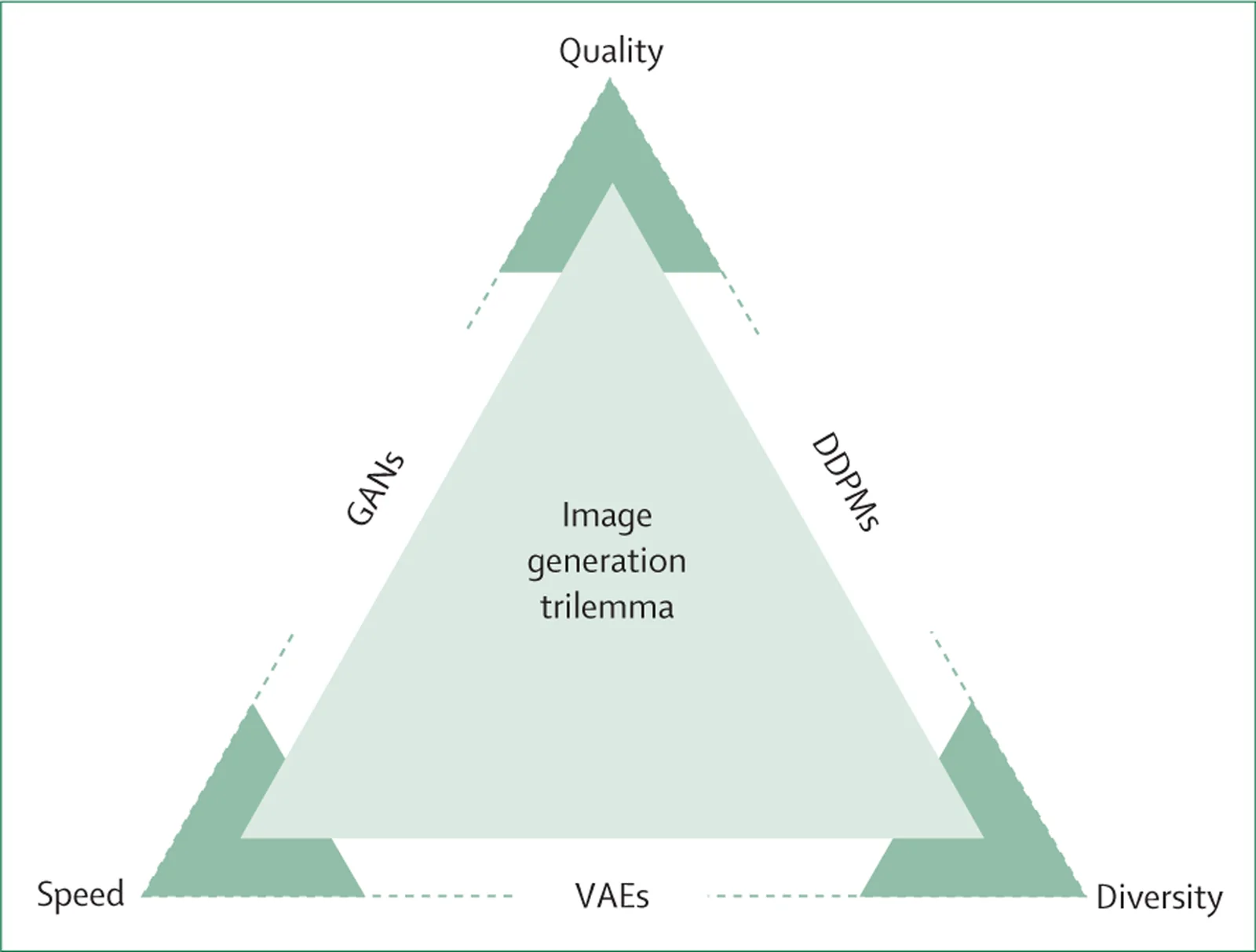
The image generation trilemma, which represents the trade-offs between three key aspects of generative models: diversity, quality, and speed VAEs excel in generating diverse samples quickly but can compromise on image quality. GANs strike a balance, providing good quality and diversity but can suffer from mode collapse, thereby restricting the diversity. DDPMs prioritise high-quality and diverse samples at the cost of a slow generation speed. DDPMs=denoising diffusion probabilistic models. GANs=generative adversarial networks. VAEs=variational autoencoders.

Statistical models encounter the generative artificial intelligence trilemma, which involves balancing high sample quality, comprehensive mode coverage, and rapid sampling rates (figure 2).^18^ VAEs are notable for their quick sampling capabilities, sometimes resulting in lower sample quality. GANs excel at generating high-quality samples but might not always capture all data variations, leading to low mode coverage, known as mode collapse. DDPMs stand out for their ability to generate samples of exceptional quality and extensive mode coverage, albeit at a slower sampling rate. End users select the generative model that matches their application of interest, balancing the desired image quality and speed. For dataset generation purposes, the priority typically shifts towards ensuring high image quality and comprehensive mode coverage, often outweighing concerns of sampling speed.

### Use cases in medical imaging

Generative models and their synthetic datasets have numerous applications in medical imaging (panel 1). One well studied use case involves supplementing or replacing real data to train deep learning models for downstream tasks such as classification or segmentation. Generated images can be conditioned on class labels (eg, presence or absence of pneumonia) or descriptive text (eg, right middle lobe consolidation). Research has shown that images generated by GANs and DDPMs can improve the performance of downstream pathology classifiers substantially.^7,19,30^ Notably, the classifier performance improves as more synthetic data are added to the real dataset. In some cases, a sufficiently large pool of generated images can match the performance benefit of real data, potentially opening new avenues for data sharing whereby synthetic data acts as a replacement of the original data.^8^ However, when training and evaluating generative models, caution is required to avoid distribution leakage (in which a patient is represented in both training and test data), which could overestimate performance improvements.^8^ Of note, repeatedly training image generation models on the output of other generative models (usually more than three iterations) risks mode collapse, which degrades the quality of the final model.^31^

Generative models also excel at image transformations. VAEs and GANs have long enabled low-dose CT image denoising, eventually reducing radiation exposure for patients.^32,33^ Of late, accelerated MRI techniques have been used to reduce the scan time by 30%.^34^ Another image-to-image transformation use case generates missing MRI sequences, enabling training of downstream algorithms requiring all four sequences: T1, T2, post-contrast T1, and FLAIR.^23,29^ DDPMs have enabled inpainting, which involves selectively adding or removing specific image parts on the basis of criteria, without altering the context. For instance, trained diffusion models can introduce brain tumour lesions in healthy brain MRIs or remove tumoural regions by drawing on an image.^24^ Such edits can enrich under-represented datasets and introduce rare conditions, such as adding brain tumours to individuals with Alzheimer’s disease. A more advanced version of the inpainting technique was developed to edit specific regions of a chest radiograph using text prompts.^26^ The resulting edited images were used to stress-test existing models—for example, removing chest tubes from pneumothorax images to evaluate classifier performance without this known confounder.^35^

### Evaluating image quality

Evaluating the quality of generated images, which determines how these synthetic images are used, is crucial. Various metrics have been proposed to quantify the quality of generated images, both in the presence and absence of ground truth references. These metrics can be broadly categorised into two groups: image metrics and text–image metrics (panel 2).

#### Image metrics

When ground truth images are available—for example, in tasks such as super resolution and denoising of medical images—traditional metrics such as structural similarity index and the peak signal-to-noise ratio can be used to measure the similarity between the generated and reference images.^36,37^ However, in the absence of ground truth—for example, in class-conditioned image generation—alternative metrics are required. For instance, classification accuracy score trains a classification model on derived medical data and evaluates its performance on real images, providing insights into the domain adaptation capabilities of the generation models.^38^

Another widely adopted metric is the inception score, which uses an inception network pretrained on ImageNet to evaluate class predictions for a set of generated samples.^39^ Fréchet inception distance (FID) compares the means and covariances of features extracted by an ImageNet-pretrained inception network between the generated and real samples.^40^ By accounting for the target distribution, FID provides a better estimate of image diversity than inception score. Several variants and improvements of FID have been proposed—eg, the kernel inception distance is a variant of FID that enables metric calculation using a small number of samples, unlike FID calculation, which requires generation of a large number of samples and is resource intensive.^41^ One limitation of these metrics is that they depend on pretrained networks, and unlike natural images, no universally accepted model for feature extraction exists in medical imaging.

#### Human evaluation

In addition to computational metrics, human evaluation remains a gold standard for assessing the quality of generated medical images. The human Turing test involves domain experts who are asked to discern between real and derived medical images.^42^ This assessment provides insights into the perceptual quality and realism of generated images, which is crucial for medical imaging, in which accuracy and fidelity are paramount. However, as perceptual quality and realism are subjective measures, a wide range of participants with different experience levels should be involved in the image evaluation process.^43^

#### Text–image metrics

Although image metrics focus solely on the visual quality of generated images, text–image metrics aim to measure the alignment between the input text and the generated image. These metrics are particularly relevant in medical image generation tasks, in which the generated images need to reflect the textual descriptions of medical conditions or anatomical structures accurately. Metrics such as contrastive language-image pretraining score (CLIPScore) and bootstrapping language-image pretraining score (BLIP-Score) measure the similarity between the input text and the generated image, quantifying the degree of alignment between the two modalities.^44,45^

Image–text matching is another crucial group of metrics for evaluating the alignment between generated medical images and their corresponding textual descriptions. Compositional quality metrics assess this alignment by decomposing the text and image into individual components and measuring their correspondence, often using object detection techniques.^46,47^ These metrics go beyond overall visual similarity and focus on accurate representation of specific anatomical structures, pathologies, or medical conditions mentioned in the text. By ensuring that the generated images convey the intended medical information accurately, compositional quality metrics can play a key role in medical education and research.

#### Health-care-specific metrics

Evaluating synthetic medical images requires metrics tailored to health-care needs, beyond general purpose tools such as the structural similarity index or FID. Efforts are underway to adapt existing metrics for medical contexts. For instance, researchers have begun replacing Image Net-pretrained models in FID with networks trained on medical datasets such as RadImageNet to create a medical FID, which captures the statistical properties of radiology images better.^48^ However, health-care-specific metrics remain an active research area as disease classifiers might rely more on local features than global features.^49^ Similarly, anatomical accuracy is being prioritised by developing measures that use segmentation tools to ensure that crucial structures (such as organs or lesions) are preserved in synthetic images.^50^ These adaptations aim to address the limitations of standard metrics, which often fail to reflect clinical relevance or diagnostic utility.

A suggested next step is to integrate clinical validation with these computational approaches. Human evaluations such as the human Turing test already involve experts distinguishing real images from synthetic ones, offering insights into the perceptual quality that is important for medical use. For text-guided image generation, metrics such as CLIPScore are being refined by using medical foundation models such as BioMedClip.^51^ Testing synthetic images in practical, clinical tasks such as training classifiers for disease detection can further highlight their utility. Combining these efforts could provide a robust, health-care-specific evaluation, thereby ensuring that synthetic images meet both technical and clinical standards for advancing medical imaging research and practice.

## Potentials and promises

Synthetic data generation and image generation models hold immense promise for the future of medical imaging research. By leveraging the power of generative models, researchers can unlock unprecedented levels of data diversity, privacy preservation, and multifunctionality, changing the way dataset creation, utilisation, and disease modelling are approached.

### Increased dataset size and diversity

One of the key advantages of generating data via statistical models lies in their ability to increase dataset size and diversity. Preliminary evidence suggests that generative models can be trained to disentangle specific associations within data, allowing for the creation of novel combinations that might not be readily available in real-world datasets.^52,53^ For instance, a model trained on brain MRI scans can generate images with varying degrees of atrophy or lesion load, independent of factors such as age or sex. Such disentanglement enables training models to detect specific pathologies without confounding the effects of other variables. As mentioned earlier, supplementing increased dataset size with generated images could lead to enhanced downstream model performance.^8^ Moreover, targeted oversampling of minoritised sociodemographic groups or patients diagnosed with rare diseases through synthetic data generation has been shown to close the fairness gap by 40%.^22^ Synthetic data generation closes this fairness gap by facilitating an increase in dataset sizes that represent the original dataset distribution for various subgroups.

### Privacy preservation

Synthetic datasets offer a privacy-preserving solution to the challenges of sharing and utilisation of data in medical research.^54^ Generative artificial intelligence anonymises sensitive patient information by generating realistic images that mimic biological characteristics of real patient data (both visually and in the model feature space) without direct replication of original data.^55^ Such anonymisation enables the creation of datasets that can be shared and analysed without compromising patient privacy, which further opens up new avenues for collaborative research and facilitates the development of robust, privacy-compliant artificial intelligence models in medical imaging.

### Versatility across tasks

Another key potential of image generation models, especially DDPMs, lies in their multifunctional nature. Generative models trained on medical images can be adapted and repurposed for various tasks beyond supplementing data; for example, features learned from an unsupervised image generation model can be leveraged for few-shot image segmentation, enabling accurate delineation of anatomical structures or pathologies with only 20 expert-annotated examples.^27^ The same model without any further training can also be used for inpainting to create diverse training samples.^56^ Similarly, generative models without any fine-tuning after initial training can be used for anomaly detection in medical images.^57,58^ This versatility extends the value of the synthetic datasets and their generator models, as a single model can be used for multiple downstream applications, streamlining research workflows, and reducing the need for task-specific data collection and model development.

### Modelling complex biological phenomena

Advanced generative models can internalise complex biological phenomena through their training procedures, enabling the intricate physiological processes to be modelled and simulated.^59^ This internalised world model can be leveraged for novel applications that extend beyond the downstream tasks discussed in the previous section. One striking example of this capability is the prediction of post-operative imaging appearances. When trained on a large corpus of paired prearthroplasty and postarthroplasty pelvic radiographs, these models generated highly realistic post-operative radiographs, simulating a well executed surgery.^28^ Remarkably, domain-expert surgeons evaluated the generated postoperative images as more robust and anatomically accurate than their real counterparts, highlighting the potential of these models in serving as virtual surgical planning tools and educational resources.^28^

Another compelling application of this internalised world model is the prediction of disease progression.^30^ For instance, when given an initial brain MRI scan and information about the patient’s treatment regimen, advanced DDPMs can generate a series of images that depict the potential progression of a brain tumour over time.^31^ By learning the complex interplay between disease characteristics, treatment effects, and biological processes, these models can provide valuable insights into patient prognosis and aid clinical decision making.^31^

## Challenges and considerations

Although derivative synthetic datasets and image generation models hold immense promise for medical imaging research, several challenges and ethical considerations need to be addressed to ensure their responsible and effective utilisation. Panel 3 summarises these challenges and proposes some future research directions to mitigate them.

### Patient privacy and data copying

Although synthetic datasets can help to preserve patient privacy by generating anonymised data, concerns regarding potential data copying still exist.^60^ If a generative model is trained on a specific dataset and can replicate images that closely resemble the original data, then the model might inadvertently reveal sensitive patient information. Copying happens when multiple copies of the image or captions are present in the dataset, which not only necessitates careful data curation,^61^ but also raises concern about the degree of anonymisation achieved in the training data and the potential for reidentification. Unlike tabular data, medical images contain patient-identifying information embedded within the pixel values, thus posing unique challenges for anonymisation. For instance, facial features in brain MRIs or distinctive anatomical markers in radiographs might enable reidentification even when explicit patient identifiers are removed.^62,63^

Researchers need to carefully assess the risk of data copying and implement measures to mitigate this concern, such as using differential privacy techniques or post-hoc data anonymisation.^64,65^ Advances made over the past 4 years in privacy evaluation metrics for synthetic data, such as membership inference attacks and similarity scores between real and generated samples, can help to quantify privacy risks. Additionally, emerging standards for synthetic content provenance, including the Coalition for Content Provenance and Authenticity (C2PA) and Google’s SynthID, have been developed to label artificial intelligence-generated content, addressing both transparency and intellectual property concerns.^66^

### Identification of source dataset and disclosure

Transparency regarding the source datasets used to train generative models is crucial in ensuring the integrity and reproducibility of research findings. However, identifying the specific training datasets can be challenging, especially when models are trained on multiple proprietary sources or when researchers use pretrained models without full knowledge of their training data.^67^ This insufficient transparency can hinder the ability to assess potential biases or limitations in the generated data. To address this gap, researchers should strive to document and disclose all source datasets used in the training process, enabling better understanding and validation of the derived data. Additionally, specific hyperparameters used for inference, specific class or prompt conditions, and every post-processing step involved in creating the synthetic dataset should be released along with the model or dataset release, to ensure reproducibility and applicability of the downstream work.^68^ Dataset documentation guidelines, such as the STANDING Together guidelines published in 2024, should be adopted for synthetic data generation models.^69^

### Interpretability and explainability

As generative models become increasingly complex, their interpretability and explainability will become more challenging. Understanding how these models learn and generate data is crucial for building trust in their outputs and ensuring their safe and reliable use in medical imaging research. Although some specific explainability methods devised for generative models exist to ensure proper understanding of input text or to add uncertainty measures to the datasets, adaptation and evaluation of these methods in medical imaging remains restricted.^70,71^

### Potential biases

The use of synthetic datasets and generative models raises important bias considerations. The potential for biases in the source datasets getting propagated or amplified in the generated data is a key concern.^72^ If the training data are biased towards some demographics, pathologies, or imaging protocols, then the resulting generated data could perpetuate these biases, leading to skewed researchfindings or discriminatory applications.^25^ For instance, historically, many medical imaging datasets have under-represented minoritised populations, resulting in artificial intelligence systems with likely differential performance levels across demographic groups.^73^ When representation is low, generative models could struggle to capture a true distribution of these under-represented groups. However, a 2023 study suggests that newer generative models can arrive at meaningful representations from as few as 20 samples when the overall dataset is sufficiently large to capture high-level features.^61^ Mitigation strategies in this case include diversity-aware sampling during training, adversarial debiasing techniques, explicit fairness constraints in model objectives, and leveraging the few-shot fine-tuning capabilities of newer generative models.^74^ Researchers need to actively assess and mitigate potential biases in the source data and regularly audit the generated data for fairness and representativeness.

## Future directions

The field of generative artificial intelligence in medical imaging is evolving rapidly, and several key areas of research and development hold promise for advancing the capabilities and applications of synthetic datasets and image generation models. One crucial direction is the development of more robust and standardised evaluation frameworks that consider the unique challenges and requirements of medical imaging, including establishment of clinically relevant metrics, benchmark datasets, and challenges concerning comparative analysis and validation of different generative models.^75^

Another important avenue is the exploration of novel architectures and training strategies, such as hybrid models combining physics-informed and statistical approaches with incorporation of domain-specific knowledge and constraints. Integration of generative models with other artificial intelligence techniques such as reinforcement and active learning could enable the creation of personalised and patient-specific datasets for precision medicine and targeted treatment planning.^76^

Addressing the ethical and regulatory challenges surrounding the use of synthetic datasets and image generation models is essential to realise their full potential, and requires collaboration among researchers, clinicians, ethicists, and policy makers to develop guidelines and best practices for responsible use, data privacy, consent, and accountability. Regulatory bodies, including the US Food and Drug Administration (FDA) and the European Medicines Agency, will play a crucial role in establishing frameworks for validating and approving synthetic data for clinical applications. Frameworks for evaluating synthetic medical imaging are already emerging, as evidenced by the FDA’s clearance of synthetic MRI technologies.^77^ These technologies were regulated as image processing software rather than as completely novel modalities, with the FDA requiring extensive clinical validation to show that the diagnostic performance of the radiologist remained equivalent when using synthetic images versus conventional images. This regulatory precedent suggests a pathway for future synthetic data technologies: proof-of-performance equivalence on standardised diagnostic tasks, rigorous clinical validation with multiple readers, and postmarket surveillance commitments to monitor for any divergence in clinical outcomes.

In conclusion, derivative synthetic datasets and image generation models have the potential to change medical imaging research and clinical practice. Addressing the challenges associated with them, establishing best practices, and investing in research and innovation can help to harness the full potential of generative artificial intelligence in improving patient care, advancing scientific discovery, and transforming the landscape of medical imaging.

## Panel 1: Use cases of synthetic imaging datasets and image generation models in medicine and their findings

We performed a literature search using the following term combinations: “synthetic data” OR “VAE” OR “GAN*” OR “diffusion model*” AND “medical imag*” OR “radiolog*” OR “dermatolog*” OR “patholog*”, and selected a representative paper matching each type of synthetic data for inclusion.

**Chambon et al (2022)**:^7^ Generating chest radiographs conditioned on input prompts

- Improved classifier performance by 5% when trained on combined synthetic and real data
- Increased classifier performance by 3% when trained solely on a larger synthetic dataset
- Enhanced text encoder representation for pneumothorax detection by 25% after fine-tuning

**Pinaya et al (2022)**:^9^ Generating 3D brain MRIs and investigating the conditioning factors

- Enabled controlled generation of realistic 3D brain MRIs with adjustable age, sex, and structural parameters
- Generated a synthetic dataset of 100 000 brain images for public use

**Frid-Adar et al (2018)**:^19^ Generating abnormal samples to tackle class imbalance in liver lesion detection on CT scans

- Increased liver lesion detection sensitivity from 78⋅6% to 85⋅7% using synthetic data augmentation
- Improved specificity from 88⋅4% to 92⋅4% with synthetic images
- Radiologists found synthetic images indistinguishable from real ones in blinded assessments

**Khosravi et al (2024)**:^8^ Using synthetic chest radiographs to supplement real images to expand the training set of pathology classifiers

- Enhanced AUROC by up to 0⋅02 in internal and external test sets with ten times synthetic data supplementation
- Synthetic-trained classifiers matched the performance of real-data models using 33–50% fewer images
- Combining real and synthetic data improved AUROC of pathology classifiers from 0⋅76 to 0⋅80 in cross-source testing

**Ktena et al (2024)**:^20^ Using synthetic images to increase the fairness of downstream classifiers on multiple modalities

- Reduced fairness gap by 44⋅6% in chest radiograph classifiers trained on synthetic and real images
- Improved out-of-distribution prediction accuracy by 7⋅7% across pathology slides
- Increased dermoscopy high-risk sensitivity by 63⋅5% and reduced fairness gaps by 7⋅5 times

**Conte et al (2021)**:^21^ Creating missing brain MRI sequences for streamlined processing

- Boosted tumour segmentation Dice coefficient from 0⋅79 to 0⋅83 with synthetic MRI sequences

**Rouzrokh et al (2022)**:^22^ Introducing and removing lesions from brain MRI slices

- Effectively inpainted (which involves selectively adding or removing specific image parts on the basis of criteria, without altering the context) tumour components, random tumours, and healthy brain tissues using DDPMs

**Khosravi et al (2024)**:^23^ Creating counterfactual pelvis radiographs from different race groups to evaluate disparities in large imaging datasets

- Identified racial disparities in prevalence of osteoarthritis between African American patients and White patients
- Highlighted dataset-scale disparities by means of synthetic counterfactual pelvis radiographs

**Pérez-García et al (2023)**:^24^ Stress-testing image classifiers by creating counterfactuals to evaluate possible shortcuts and their effect on model performance

- Generated counterfactual datasets simulating acquisition, manifestation, and population shifts
- COVID-19 classifier accuracy dropped from 99⋅1% to 5⋅5% when COVID-19 features were removed
- Pneumothorax classifier accuracy dropped from 93⋅3% to 17⋅9% when chest tubes were artificially removed

**Khosravi et al (2023)**:^25^ Using internal features of generative models for label-efficient pelvis radiograph segmentation

- Enhanced pelvis radiograph segmentation accuracy by 0⋅30–0⋅32 points using generative model features, using only 20 annotated samples

**Rouzrokh et al (2024)**:^26^ Creating synthetic postoperative images of patients undergoing total hip arthroplasty

- Produced synthetic postoperative hip radiographs with a mean acetabular angle of 39⋅9° (±4⋅6), 99% within safe zones
- Synthetic radiographs scored higher validity (9⋅0±0⋅7) than real ones (7⋅9±1⋅1)

**Yuan et al (2024)**:^27^ Imputing missing 3D brain MRIs in Alzheimer’s longitudinal studies conditioned on past or future scans

- Achieved SSIM of 0⋅895 (with skull) and 0⋅983 (skull removed), outperforming autoencoders (0⋅74 for with skull and 0⋅91 for skull removed) and naive methods (0⋅70 for with skull and 0⋅89 for skull removed)
- Reduced volumetric error rates from 0⋅14 (using conventional methods) to 0⋅05

**Kyung et al (2024)**:^28^ Forecasting chest radiograph morphology on the basis of electronic health record data

- Achieved a weighted macro AUROC of 0⋅72 in predicting future chest x-ray pathologies, outperforming tabular-only classifiers and previous label baselines
- Maintained sex (AUROC 0⋅96) and age (0⋅45) correlations in synthetic images

**Liu et al (2025)**:^29^ Forecasting tumour growth on the basis of baseline tumour characteristics and treatment plan

- SSIM of 0⋅92 and PSNR of 29⋅0 for multiparametric MRI generation, outperforming baseline models without treatment-aware conditioning
- Generated MRI quality remained high across different treatment-day ranges, with SSIM ranging from 0⋅88 to 0⋅94 depending on the treatment phase
- Tumour growth predictions were most reliable within a 4-month window, with the Dice similarity coefficient dropping from 0⋅85 to 0⋅46 as the time interval extended from 0⋅5 months to greater than 24 months

3D=three dimensional. AUROC=area under the receiver operating characteristic curve. DDPMs=denoising diffusion probabilistic models. PSNR=peak signal-to-noise ratio. SSIM=structural similarity index.

## Panel 2: Summary of image quality metrics based on use case for medical image generation

### Image super resolution, denoising, and inpainting

- SSIM: assesses structural similarity between generated and reference images by considering luminance, contrast, and structure
- PSNR: measures the ratio between the maximum possible power of a signal and the power of corrupting noise between generated and reference images

### Class-conditioned and unconditional image generation

- IS: compares class predictions and diversity of generated samples using an inception network pretrained on ImageNet
- FID: compares means and covariances of features extracted from generated and target distributions using an inception network pretrained on ImageNet
- KID: computes squared MMD between inception representations of generated and target distributions using an inception network pretrained on ImageNet

### Domain adaptation, and class-conditioned image generation

- CAS: uses a classifier trained on derived medical images and evaluates performance on real images

### Perceptual quality assessment, and realism evaluation

- Human Turing test: medical experts discern between real and derived images

### Image generation from textual descriptions

- Segmentation-based metrics: volumetric analysis of different organs on generated images and comparing them with the input condition
- CLIPScore: computes cosine similarity between CLIP embeddings of text descriptions and generated images
- BLIPScore: computes cosine similarity between BLIP embeddings of text descriptions and generated images
- LLMScore: leverages an LLM for creating a detailed caption at the level of an image and different objects and compares the generated caption with the input text descriptions

BLIP=bootstrapping language-image pretraining. CAS=classification accuracy score. CLIP=contrastive language-image pretraining. FID=Fréchet inception distance. IS=inception score. KID=kernel inception distance. LLM=large language model. MMD=maximum mean discrepancy. PSNR=peak signal-to-noise ratio. SSIM=structural similarity index.

## Panel 3: Summary of challenges, considerations, and future research directions for generative artificial intelligence and synthetic datasets in medical imaging

### Data copying

Generative models can inadvertently reveal sensitive patient information when they reproduce images that closely resemble the original data. Future research directions:

- Creating metrics to quantify the privacy risk of generated images
- Developing post-hoc data anonymisation methods
- Investigating the trade-off between image quality and privacy preservation

### Identification of source dataset

Identifying specific datasets used to train generative models can be challenging, hindering the assessment of potential biases or limitations in the generated data. Future research directions:

- Creating standardised reporting guidelines for synthetic medical imaging datasets
- Developing techniques for dataset fingerprinting in generative models
- Creating trusted third-party validation services for synthetic medical datasets
- Exploring methods for reverse-engineering model-training data

### Interpretability and explainability

The complex nature of generative models makes understanding how these models learn and generate data a challenge. This understanding is necessary to build trust in the model outputs.

Future research directions:

- Implementing uncertainty quantification methods for stochastic prediction models
- Creating clinically relevant interpretability metrics
- Developing interactive visualisation tools for clinicians to explore model decisions
- Investigating the integration of domain knowledge into model explanations

### Potential biases

Biases in the source datasets could be propagated or amplified in the generated data, leading to skewed research findings or discriminatory applications. Future research directions:

- Creating benchmarks for evaluating fairness in medical imaging generative models
- Establishing multi-institution collaboratives to create demographically balanced training data
- Investigating the effect of data augmentation on bias reduction
